# An Efficient Method for Optimizing HPC-FRP Retrofit Systems of Flexural Strengthened One-Way Continuous Slabs Based on ACI 440.2R

**DOI:** 10.3390/ma15238430

**Published:** 2022-11-26

**Authors:** Huy Q. Nguyen, Kijae Yang, Jung J. Kim

**Affiliations:** 1 Department of Civil Engineering, Kyungnam University, Changwon-si 51767, Republic of Korea; 2 Corporate Partnership Center, Korea Authority of Land & Infrastructure Safety, Jinju-si 52856, Republic of Korea

**Keywords:** FRP, continuous RC slab, retrofit, strengthen, optimal design

## Abstract

An innovative retrofit system consisting of fiber-reinforced polymers (FRP) and high-performance concrete (HPC) considering the difficulty of the accessibility and installation of FRP on the underside of reinforced concrete (RC) slabs was found to be efficient in the flexural strengthening of existing RC slabs. It is important to note that continuous slabs using the FRP-HPC retrofit systems are less effective in exploiting FRP tensile strength and can cause sudden failure once excessively enhanced flexural strength exceeds shear strength. A design method to ensure ductile failure mode was also proposed for strengthened continuous RC slabs in the previous literature. Thus, it is necessary to optimize retrofit systems in terms of mechanical performance aspects to improve the efficiency of retrofitted slabs in serviceability. This study proposes a design method for optimizing the strength of materials and inducing ductile failure of continuous slab retrofitting FRP-HPC systems. The proposed approach demonstrated its effectiveness for strengthening a continuous RC slab with various FRP-HPC retrofit systems through a case study. The results show that the design factored load in the serviceability limit state does not change appreciably from a decrease in carbon fiber-reinforced polymers (CFRP) of 38%; the design factored load decreased only by 9% and the ultimate failure load by 13% while reducing CFRP by 20% and HPC by 25%.

## 1. Introduction

Structural strengthening has seen tremendous advancements in materials, methods, and techniques in the last few decades. Enhancing the lifecycle of existing RC structures and reducing environmental impact has become an attractive topic in the structural engineering community [1,2]. The strengthening of existing civil engineering infrastructure with externally bonded FRP has emerged as one of today’s state-of-the-art techniques for rehabilitating and improving the load carrying capacity of existing RC structures [3,4,5,6]. Of course, concrete substrates of existing RC structures should also possess the required strength to develop the design stresses of the FRP system through the bond regarding flexure or shear strengthening [7]. The acceptance of FRP materials in restoring and strengthening damaged RC structures due to their low weight, high tensile strength, immunity to corrosion, and unlimited sizes is recognized widely in the available literature [8,9,10,11,12]. In addition, novel methods and techniques for strengthened RC structures using FRP composite materials have also been developed in proportion to their growth in the level of popularity [13,14,15,16,17].

**Figure 1:**
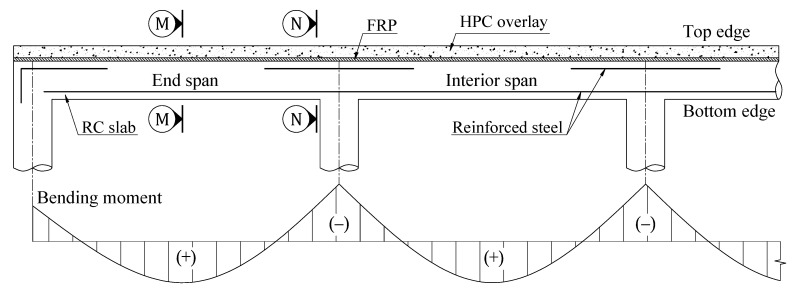
An innovative HPC-FRP hybrid retrofit system for strengthening continuous RC slab.

Conventionally, methods of strengthening RC slabs by attaching FRP to tensile zones to maximize the high tensile strength of composite materials have gained wide application in practice [18,19,20]. Unfortunately, it can be impossible to acquire a well-prepared concrete surface in some cases due to the difficulty of the accessibility and installation of FRP on the underside of the RC slabs [21]. Furthermore, the ductility reduction due to the intrinsic bond of the FRP-to-concrete interface leading to brittle failure is one of the notable drawbacks of strengthening RC structures using FRP [22,23,24]. The structural ductility factor should be considered the vital requirement for preventing brittle shear failure and warning of forthcoming failure for practical designs [25,26,27]. With a focus on overcoming the drawbacks of typical strengthening techniques, an innovative hybrid retrofit system using CFRP with HPC overlay on the top surface of the existing RC slab was developed, instead of taking advantage of the high tensile strength of CFRP, as shown in Figure 1. Previous studies have confirmed the efficiency of retrofit systems in improving strength and ductility, along with overcoming logistical challenges without complex engineering requirements [21,28,29].

For post-strengthened slabs, additional strength should be limited to avoid sudden failure, which could result from an excessive enhancement in flexural strength over shear strength. Thus, previous studies have developed novel failure mode classification and failure limits for continuous RC slabs based on their shear- and moment-carrying capacities [30,31]. A calculation method for retrofitted slabs to prevent brittle failure and induce ductile failure was also recommended. Regardless, the design methodology also has restrictions in considering the demand for strengthening each different location appropriately to optimize the strength of the constituent materials consisting of CFRP and HPC.

Although design guidelines for FRP strengthening structures have been reported, optimizing continuous RC slabs using retrofit systems has not been considered comprehensively [32,33,34,35]. It is possible, for example, for the mid-span sections to fail before the support sections reach the limit state or vice versa. These guidelines, although applicable, are not appropriate for optimizing the bearing capacity of retrofitted slabs. In addition, these standards were also not developed to ensure ductile failure for strengthened slabs. Optimizing materials’ strength and inducing ductile failure of retrofitted slabs should be performed in the design of retrofit systems, resulting in reduced cost and more safety [36,37,38]. It will also partially overcome the shortcoming of retrofit systems, which cannot take advantage of the high tensile strength of FRP.

In this study, the flexural failure limits for the interior and end spans of continuous RC slabs following their moment and shear capacities are presented. The retrofitting mechanism for negative and positive moment sections of slabs is explained. Optimal criteria and an efficient design procedure for flexural strengthened continuous RC slabs using FRP-HPC retrofit systems are proposed based on ACI 440.2R. Several approaches are considered to develop potential scenarios for strengthening solutions. An innovative method of determining the amount of CFRP and HPC for optimizing the strength of materials and inducing ductile failure of slabs by applying this strengthened technique is illustrated clearly through a case study. The advantages and disadvantages of the proposed method are also discussed based on the obtained results.

## 2. Theoretical Background

### 2.1. Failure Limits

According to previous works [30,31], the failure limits of one-way continuous slabs in frames subjected to uniformly distributed loads are defined. The slab’s shear and moment carrying capacities are used to predict the failure mode and ultimate failure load. In a frame, the distribution of moments depends on the flexural rigidity of members and supporting columns. The shears at the end of the continuous slab are taken as the simple slab shear, except at the exterior face of the first interior support, where the shear force should be higher because it has greater fixity. The maximum positive and negative moments and shears due to uniformly distributed load are calculated as follows [39]:

(1) $$M_{u}=C_{m}\left(w_{u}l_{n}^{2}\right)$$

(2) $$V_{u}=C_{v}\left(\frac{w_{u}l_{n}^{}}{2}\right)$$

**Figure 2:**
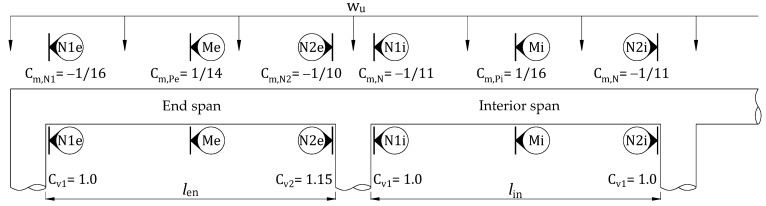
Shear and moment coefficients for continuous RC slabs with column supports, according to ACI 318M.

Considering two adjacent spans of approximately equal length or a longer span not exceeding 1.2 times the shorter, ACI 318M recommends approximate moment and shear coefficients to estimate reasonable moment and shear envelopes for a one-way slab with columns for support [40], as shown in Figure 2.

**Figure 3:**
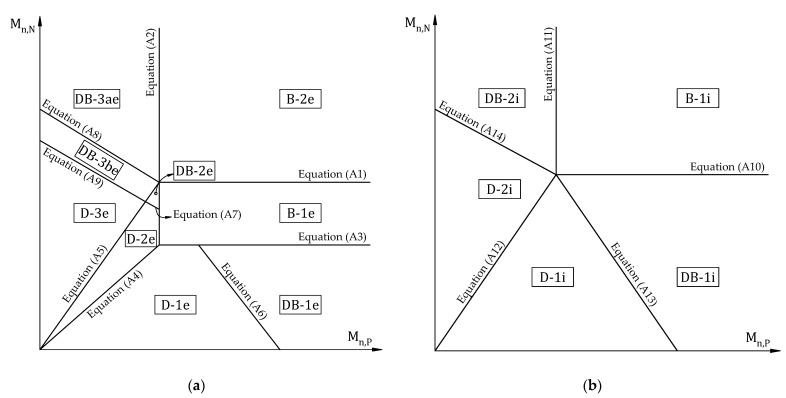
Different failure modes according to moment and shear capacities of continuous RC slabs for (**a**) end span and (**b**) interior span.

Previous studies also proposed limit equations to divide distinct regions corresponding to failure modes, described in Appendix A. Based on this, the different failure modes for the end and interior spans of the continuous slab are depicted in Figure 3. Failure modes are also classified based on the order of forming plastic hinges and failure types. The different failure modes for the end and interior span of continuous slabs are summarized in Table 1.

The superposition method considering plastic redistribution of the strengthened slab is applied to calculate the ultimate failure loads. For the end span, the ultimate failure loads for failure modes D-1e, D-2e, and D-3e are calculated from the expressions:

- Failure mode D-1e

(3) $$w_{f}=\phi_{f}\frac{8}{l_{\mathrm{en}}^{2}}\left(M_{n,\mathrm{Pe}}+M_{n,\mathrm{Ne}}\frac{\left(1/8-C_{m,\mathrm{Pe}}\right)}{C_{m,N2}}\right)$$

- Failure mode D-2e

(4) $$w_{f}=\phi_{f}\frac{4}{l_{\mathrm{en}}^{2}}\left(M_{n,\mathrm{Pe}}+M_{n,\mathrm{Ne}}\frac{\left(1/4+C_{m,N2}-C_{m,N1}-C_{m,\mathrm{Pe}}\right)}{C_{m,N2}}\right)$$

- Failure mode D-3e

(5) $$w_{f}=\phi_{f}\frac{4}{l_{\mathrm{en}}^{2}}\left(M_{n,\mathrm{Pe}}\frac{\left(1/4-C_{m,N1}\right)}{C_{m,\mathrm{Pe}}}+M_{n,\mathrm{Ne}}\right)$$

The ultimate failure loads for failure modes DB-1e, DB-2e, DB-3ae, DB-3be, B-1e, and B-2e are calculated as:

(6) $$w_{f}=\phi_{v}\frac{2V_{n}}{C_{v2}l_{\mathrm{en}}}$$

For interior span, it is possible to estimate the ultimate failure loads for failure modes D-1i and D-2i as follows:

- Failure mode D-1i

(7) $$w_{f}=\phi_{f}\frac{8}{l_{\mathrm{in}}^{2}}\left(M_{n,\mathrm{Pi}}+M_{n,\mathrm{Ni}}\frac{\left(1/8-C_{m,\mathrm{Pi}}\right)}{C_{m,N}}\right)$$

- Failure mode D-2i

(8) $$w_{f}=\phi_{f}\frac{8}{l_{\mathrm{in}}^{2}}\left(M_{n,\mathrm{Pi}}\frac{\left(1/8-C_{m,N}\right)}{C_{m,\mathrm{Pi}}}+M_{n,\mathrm{Ni}}\right)$$

The ultimate failure loads for failure modes DB-1i, DB-2i, and B-1i can be estimated as follows:

(9) $$w_{f}=\phi_{v}\frac{2V_{n}}{C_{v1}l_{\mathrm{in}}}$$

**Figure 4:**
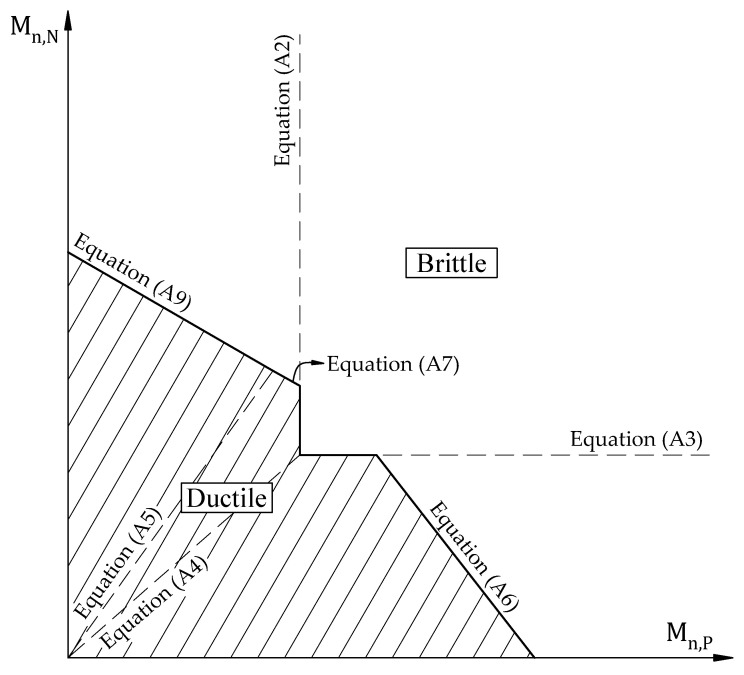
Limit failure regions for a continuous slab with the same clear span.

In case the end and interior span have the same length and structure, it is worth noticing that failure types would be determined to follow the limits of the end span, as shown in Figure 4. Otherwise, it is decided through corresponding limit equations, as earlier mentioned.

### 2.2. Retrofitting System

**Figure 5:**
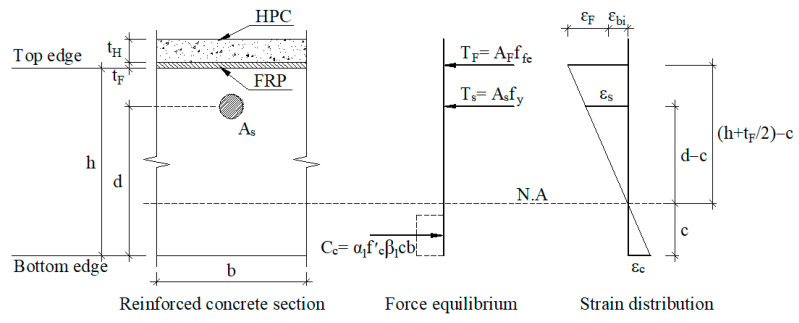
Retrofitting mechanism for negative moment sections (refer to section “N-N” of Figure 1).

A hybrid retrofit system of FRP and HPC is installed on top of the existing slab to enhance its strength, as shown in Figure 1. According to ACI 440.2R, the retrofitting mechanism for negative and positive moments of the retrofitted slab was derived based on the sectional compressive force in HPC and the sectional tensile forces in the steel and FRP. For negative moment sections, retrofitting for RC flexural members as section N-N of Figure 1 can be done in a conventional way. The retrofitting mechanism for the FRP-HPC system is estimated based on stress and strain compatibility, as shown in Figure 5. The equilibrium equations must be solved iteratively due to the existence of two sectional forces in steel and FRP, besides the possibility of different failure modes. Assuming concrete with an ultimate strain of 0.003 and steel with yield stress (f_y_), the force and moment equilibrium equations based on strain compatibility can be established from the expressions.

(10) $$\alpha_{1}{\;f^{\prime }}_{c}\beta_{1}\mathrm{cb}=A_{s}f_{y}+A_{F}f_{\mathrm{fe}}$$

(11) $$\phi_{f}M_{n}=\phi_{f}\left[A_{s}f_{y}\left(d-\frac{\beta_{1}c}{2}\right)+\psi_{f}A_{F}f_{\mathrm{fe}}\left(d_{f}-\frac{\beta_{1}c}{2}\right)\right]$$

**Figure 6:**
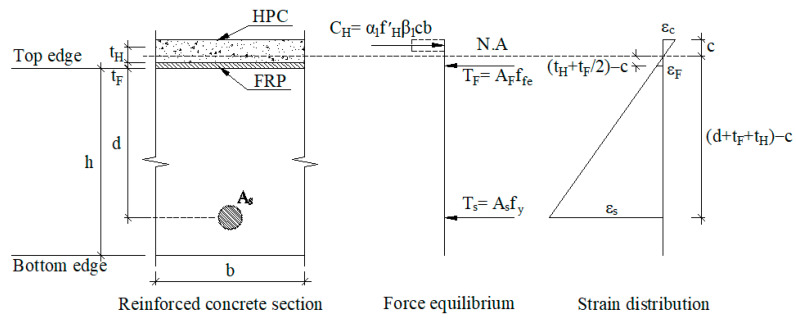
Retrofitting mechanism for positive moment sections (refer to section “M-M” of Figure 1).

For the positive moment sections, the HPC overlay on the top of the slab as section M-M of Figure 1 must have enough thickness and compressive strength to pull the neutral axis towards the overlay zone leading to FRP in tension at failure. The rationale for the retrofitting mechanism is similar to that for negative moment sections, as shown in Figure 6. Based on strain compatibility, the two governing equilibrium equations can be derived as follows:

(12) $$\alpha_{1}{\;f^{\prime }}_{H}\beta_{1}\mathrm{cb}=A_{s}f_{y}+A_{F}f_{\mathrm{fe}}$$

(13) $$\phi_{f}M_{n}=\phi_{f}\left[A_{s}f_{y}\left(d+t_{H}+t_{F}-\frac{\beta_{1}c}{2}\right)+\psi_{f}A_{F}f_{\mathrm{fe}}\left(t_{H}+\frac{t_{F}}{2}-\frac{\beta_{1}c}{2}\right)\right]$$

## 3. Optimal Criteria and Design Procedure

### 3.1. Optimal Design Criteria

The successful design of a composite structure demands efficiency and safety during operation. Thus, optimizing material usability and preventing sudden failure for the retrofitted slabs are considered essential criteria in the optimal design procedure. The enhanced efficiency of the retrofit system would stem from the high compressive strength of the HPC overlay and the high tensile strength of FRP. The HPC overlay significantly enhances flexural strength at the mid-span section and shear strength at the support. On the other hand, the retrofit system does not focus on exploiting the high tensile strength of FRP at the mid-span section due to its location near the neutral axis. Consequently, FRP contributes a relatively small amount to flexural strength at the mid-span section, whereas it is the main factor in improving flexural strength at support.

For retrofitted slabs, overly thick FRP will result in an excessive enhancement of flexural strength over shear strength at support, resulting in shear failure. A too-thick overlay can excessively improve the mid-span flexural strength over the support and increase the slab’s self-weight, which does not take advantage of the structure carrying capacity. Ideally, the moment-carrying-capacity ratio of the mid-span section to the support section should be equivalent to the corresponding proportion of factored moments. For symmetric continuous slabs, the positive to negative moment ratios at the end and interior spans subjected to a uniform distributed load can be computed using ACI 318M as follows:

(14) $$\frac{M_{n,\mathrm{Pe}}}{M_{n,N1e}}=\frac{C_{m,\mathrm{Pe}}}{C_{m,N1}}=\frac{1/14}{1/16}=1.14$$

(15) $$\frac{M_{n,\mathrm{Pe}}}{M_{n,N2e}}=\frac{C_{m,\mathrm{Pe}}}{C_{m,N2}}=\frac{1/14}{1/10}=0.71$$

(16) $$\frac{M_{n,\mathrm{Pi}}}{M_{n,N1i}}=\frac{M_{n,\mathrm{Pi}}}{M_{n,N2i}}=\frac{C_{m,\mathrm{Pi}}}{C_{m,N}}=\frac{1/16}{1/11}=0.69$$

The ratios of positive and negative factored moments range from 0.69 to 1.14. Nevertheless, the moment ratio of 1.14 of Equation (14) is not a typical value for a continuous multi-spans RC slab because it is only related to the N1e section of the end span. The average moment ratio of 0.7, derived from Equations (15) and (16), should be used to optimize RC slab performance. The design approach based on failure limit methodology can achieve ductile failure and the desired moment ratio for a strengthened slab with a retrofit system by adjusting the increase of positive and negative moment carrying capacity separately. In addition to meeting the guidelines of ACI committee 440, an optimal retrofit system can be founded once the conditions for ductile failure and optimal moment ratio are satisfied.

### 3.2. Design Procedure and Flowchart

**Figure 7:**
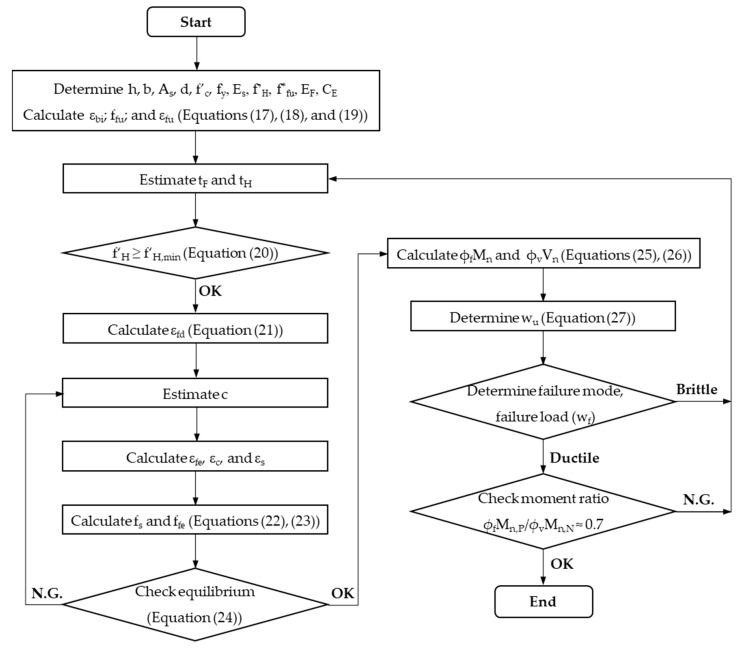
Design procedure flowchart for optimizing retrofit system.

The thicknesses of FRP and HPC are considered adjustable variables. The long-term effect of service load and different environmental conditions are not evaluated in this case. The design procedure using the proposed optimal criteria and ACI 440.2R, depicted in Figure 7, involves the following steps:

(0) Determine the known design parameters of the existing RC slab and retrofit system (i.e., h, b, A_s_, d, f’_c_, f_y_, E_s_, f’_H_, f^*^_fu_, E_F_, C_E_). Then calculate the ultimate strength (f_fu_) and strain of FRP (ε_fu_) and the existing state of strain (ε_bi_) at support from the expressions.

(17) $$f_{\mathrm{fu}}=C_{E}f_{\mathrm{fu}}^{*}$$

(18) $$\varepsilon_{\mathrm{fu}}=\frac{f_{\mathrm{fu}}^{}}{E_{F}}$$

(19) $$\varepsilon_{\mathrm{bi}}=\frac{M_{D,N2}\left(h-\mathrm{kd}\right)}{I_{\mathrm{cr}}E_{c}}$$

(1) Estimate thicknesses of FRP (t_F_) and HPC overlay (t_H_)
(2) Check HPC strength to ensure that CFRP holds tension at the failure state at the mid-span section using Equation (20) [21]. If the calculated f′_H,min_ is equal to or less than f′_H_, go to the next step; otherwise, return to step 1.

(20) $${\;f^{\prime }}_{H,\min}=\max\left[\frac{\varepsilon_{\mathrm{cu}}E_{F}}{1.445}{\left(\frac{t_{F}}{t_{H}}\right)}^{2}+\frac{f_{y}\left(A_{s}/b\right)}{0.7225t_{H}};0.15{\;f^{\prime }}_{c}+\frac{\varepsilon_{\mathrm{cu}}E_{F}}{1.7}{\left(\frac{t_{F}}{t_{H}}\right)}^{2}+\frac{f_{y}\left(A_{s}/b\right)}{0.85t_{H}}\right]\leq {\;f^{\prime }}_{H}$$

(3) Calculate the design strain of FRP (ε_fd_) at the support section as follows:

(21) $$\varepsilon_{\mathrm{fd}}=0.41\sqrt{\frac{{f^{\prime }}_{c}}{{\mathrm{nE}}_{F}t_{F}}}\leq 0{.9\varepsilon }_{\mathrm{fu}}$$

(4) Estimate the neutral axis depth with a reasonable initial value of 0.2d.
(5) Calculate the strains of FRP (ε_fe_), concrete (ε_c_), and tension reinforcing steel (ε_s_) using similar triangles based on strain compatibility mentioned in Figure 5 and Figure 6.
(6) Calculate the stresses in tension in reinforcing steel (f_s_) and FRP (f_fe_) as:

(22) $$f_{s}=\varepsilon_{s}E_{s}\leq f_{y}$$

(23) $$f_{\mathrm{fe}}=\varepsilon_{\mathrm{fe}}E_{F}\leq f_{\mathrm{fu}}$$

(7) Check the neutral axis depth for force equilibrium using c determined in Equation (24) compared with the assumed value in step 5. If the force equilibrium condition is satisfied, go to the next step; otherwise, return to step 4.

(24) $$c=\frac{A_{s}f_{s}+A_{F}f_{\mathrm{fe}}}{\alpha_{1}{\;f^{\prime }}_{c}{\;\beta }_{1}b}$$

In the case of concrete strain (ε_c_) reaching the ultimate value (ε_cu_), α_1_ and β_1_ can be calculated using ACI 318M. By contrast, these values should be calculated based on the Whitney stress block, as recommended by ACI 440.2R.

(8) Calculate design flexural and shear strengths from Equations (25) and (26):

(25) $$\phi_{f}M_{n}=\phi_{f}\left(M_{\mathrm{ns}}+\psi_{f}M_{\mathrm{nf}}\right)$$

(26) $$\phi_{v}V_{n}=\phi_{v}\left(d\sqrt{{\;f^{\prime }}_{c}}+t_{H}\sqrt{{\;f^{\prime }}_{H}}\right)\frac{b}{6}$$

(9) Determine the design factored load using Equations (27)–(29), derived from Equations (1) and (2):

(27) $$w_{u}=\min\left(w_{u,M},w_{u,V}\right)$$

(28) $$w_{u,M}=\frac{\phi_{f}M_{n}}{C_{m}l_{n}^{2}}$$

(29) $$w_{u,V}=\frac{\phi_{v}V_{n}}{C_{v}l_{n}^{}}$$

(10) Determine the failure mode corresponding to the ultimate failure load based on the proposed failure limit. If the retrofitted slab fails in ductile, go to the next step; otherwise, return to step 1.
(11) If the desirable moment ratio (*ϕ*_f_M_n,P_/*ϕ*_v_M_n,N_) is approximately 0.7, the optimal design solution for the retrofit system is achieved; otherwise, re-estimate t_F_ and t_H_.

## 4. Case Study

The rectangular RC continuous slab with the same clear span of 2.75 m subjected to uniformly distributed load is considered for a case study. As mentioned above, the failure mode of the end span governs corresponding to the moments and shears coefficients as shown in Figure 2, where C_m,N1e_ = 1/16, C_m,N2e_ = 1/10, C_m,Pe_ = 1/14, C_v1_ = 1, and C_v2_ = 1.15. The environment reduction factor for a retrofit system with CFRP overlaid by HPC (C_E_) is equal to 0.95. The strength reduction factors *ϕ*_f_, *ϕ*_v_, and *ψ*_f_ are 0.9, 0.75, and 0.85, respectively [7]. The CFRP and overlay thickness of the retrofit system are assumed as design variables, which can be adjusted to induce ductile failure and optimize the performance of a retrofitted slab based on the proposed procedure. Dimensions and material properties of the existing RC slab are provided in Table 2. The mechanical properties of the retrofit system are presented in Table 3. The design procedure considers the reliability factor for debonding CFRP, as recommended by ACI 440.2R. Besides that, a retrofit system should also include shear anchors to maintain integrity until reaching the ultimate carrying capacity. The effectiveness of shear anchors in the retrofit system was confirmed in the previous literature [28]. The preliminary calculation for the control slab and retrofit system is shown in Table 4.

## 5. Results and Discussions

**Figure 8:**
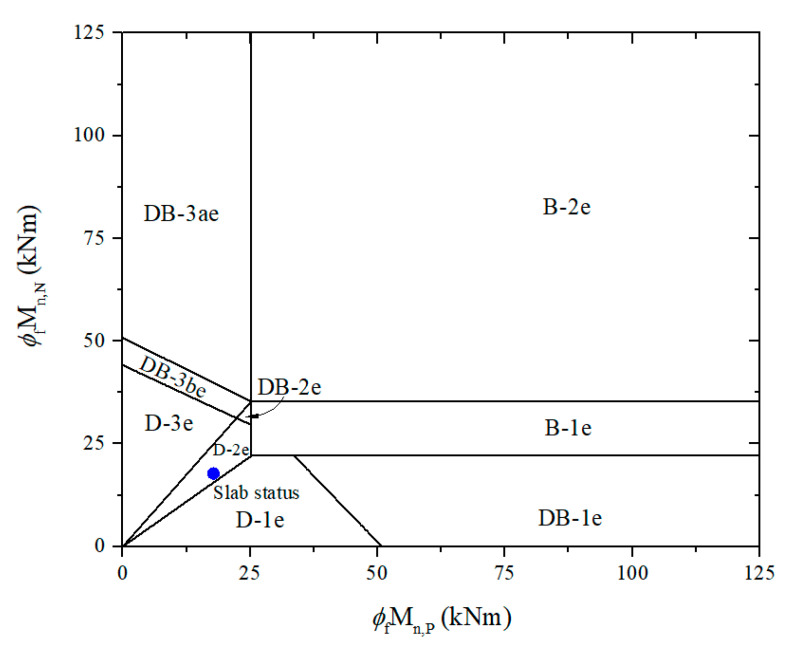
Establish failure limits and predict slab status based on moment carrying capacities for the control slab.

**Figure 9:**
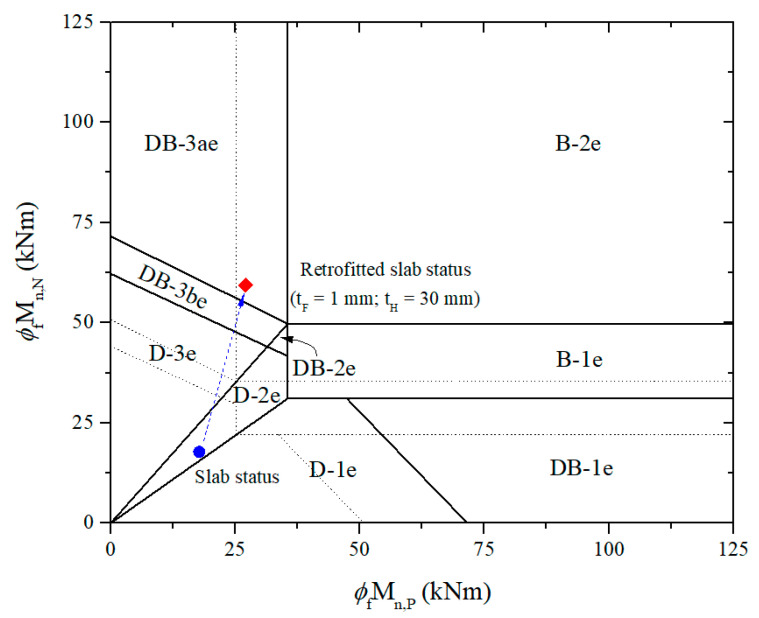
Establish failure limits and predict slab status based on moment carrying capacities for the retrofitted slab.

According to Table 4, the design factored load of the control slab is estimated as 23.6 kN/m, while the ultimate failure load is also expected at 29.8 kN/m with failure mode D-2e, as shown in Figure 8. Besides defining CFRP’s ultimate strength and debonding failure strain, the initial calculation related to the existing strain is considered only for the N-2 section, where the highest internal force is confirmed. For analysis of the retrofitted slab, the thicknesses of FRP and HPC are initially assumed to be 1 mm and 30 mm, respectively, as shown in Table 5. The design flexural and shear strengths are determined after force equilibrium is satisfied via iterative calculation. The failure mode of the retrofitted slab is named DB-3ae according to the proposed failure limit, as shown in Figure 9. The first plastic hinge will be formed at mid-span with the design factored load of 50.1 kN/m before failure in shear at the N-2 section with the ultimate failure load of 65.9 kN/m. Brittle failure is not the desired effect, even though the design factored load and ultimate failure load are higher than the control slab by 2.12 and 2.21 times, respectively.

**Figure 10:**
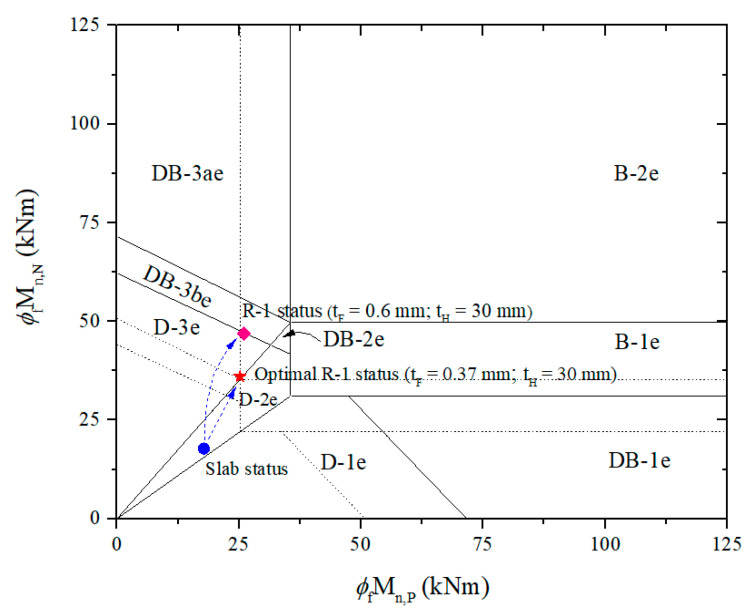
Establish failure limits and predict retrofitted slab status based on moment carrying capacities using the R-1 system.

The retrofit system is optimized by varying the thicknesses of CFRP or HPC to obtain ductile failure mode and desirable moment ratio. A similar calculation process, the R-1 system with only adjustable CFRP thickness, is considered a solution, as shown in Table 6. Figure 10 reveals that the strengthened slab can be failed in ductile failure mode D-3e with the ultimate failure load of 60.9 kN/m by using 0.6 mm-thick CFRP laminate, increased 2.04 times compared to the failure load of the control RC slab. Nonetheless, the positive-to-negative moment ratio of 0.55 may cause the mid-span section to fail before the support section. CFRP thickness should be iterated until the moment ratio is approximately 0.7, which can be met at 0.37 mm thick, resulting in failure mode D-3e with the design factored load of 46.7 kN/m and the ultimate failure load of 54 kN/m. Compared to the optimized and unoptimized retrofit systems, the former decreases CFRP by 38%, only resulting in a reduced 3% of the design factored load and 11% of the ultimate failure load. For this case, the moment carrying capacity at mid-span controlling the possibility of failure is almost unchanged by a 3% decrease, whereas it fell remarkably by 23% at the support sections. As a result, the optimal retrofit system can be determined with the mid-span and support section simultaneous failures, along with considerable savings in CFRP, while the bearing capacity almost remains unchanged.

**Figure 11:**
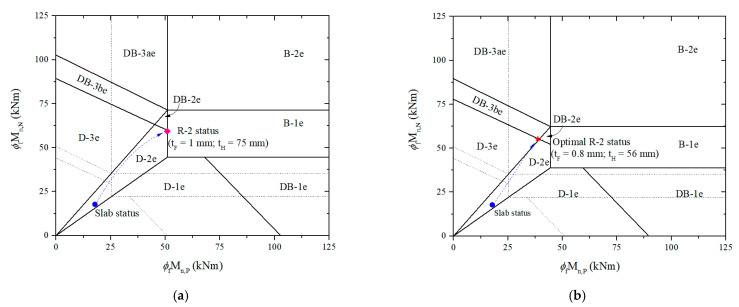
Establish failure limits and predict retrofitted slab status based on moment carrying capacities using the R-2 system, considering (**a**) ductile failure; (**b**) ductile failure and optimal moment ratio.

It is noticeable that Table 6 also shows a second alternative approach called the R-2 system with additionally adjusted HPC thickness. According to Figure 11a, ductile failure mode D-2e with the ultimate failure load of 94.9 kN/m can be obtained with thicknesses HPC of 75 mm and CFRP of 1 mm, leading to an increase of 3.18 times over the existing slab’s failure load. Nevertheless, the positive-to-negative moment ratio of 0.86 can lead to the support section failing before the mid-span section reaches its critical point. Once the moment ratio exceeds 0.7, along with CFRP being kept constant, the higher the HPC thickness, the higher the moment ratio, leading to the inability to optimize the moment ratio. Consequently, CFRP and HPC thicknesses should be adjusted simultaneously to obtain the moment ratio of 0.7. It is possible to optimize the retrofit system with 0.8 mm and 56 mm CFRP and HPC thicknesses, respectively. The failure mode is D-3e, with the ultimate failure load of 83 kN/m, as shown in Figure 11b. Compared to the unoptimized retrofit system, the optimized retrofit system decreased CFRP by 20% and HPC by 25%. Nonetheless, w_u_ and w_f_ were only reduced by 9% and 13%, respectively. In this case, the moment carrying capacity at the supports that govern probable failures did not change substantially, with a decrease of 7%, whereas in the mid-span section, it dropped significantly, by 24%. Accordingly, a retrofit system is optimized with significant CFRP and HPC savings without a noticeable change in load carrying capacities.

In this study, the prediction of flexural and shear carrying capacities of strengthening slabs with retrofit systems is shown to be in good agreement with the previous literature [28,29,30,31]. Additionally, civil engineers, especially the authors mentioned in this topic, have also long been interested in optimizing the strength of materials for more efficient workability of structures, resulting in cutting construction costs. As a result, evaluating the performance of optimized versus non-optimized retrofit systems is of particular interest in the present work. Optimized retrofit systems require far fewer resources but still provide significant efficiency in strengthening RC slabs. The strengthened slab capacities using different retrofit systems are summarized in Table 7. In addition, concrete overlays are not required to be high strength to generate tension in CFRP based on the analysis in step 2, as clarified by Mosallam et al. [21]. However, HPC is still recommended to increase flexural strength and avoid potential shear failures.

## 6. Conclusions

This paper presents the efficient design procedure for strengthening continuous RC slabs using innovative FRP- HPC hybrid retrofit systems based on ACI 440.2R. The different retrofit systems are evaluated for their pros and cons in developing possible strategies for strengthening RC slabs. The efficiency of the proposed approach involving determining the amount of CFRP and HPC to optimize the strength of materials and ensure the ductile failure of slabs using retrofit systems is demonstrated through the case study. Based on the obtained results, the following conclusion can be drawn:

The additional flexure and shear of strengthened slabs using retrofit systems are greatly influenced by the thicknesses of CFRP and overlay.

Quantitative CFRP can be adjusted separately or in parallel with HPC to optimize the retrofit system depending on the demand to improve flexural moment and shear strengths. At mid-span, the additional flexural and shear strength are notably affected by the HPC overlay, whereas at the support, they are individually governed by CFRP and overlay HPC, respectively. In case the appropriate thickness of CFRP laminate is not available, discrete CFRP strips can also be recommended.

The outcomes of the study indicated that a 38% reduction in CFRP does not significantly impact the design factored load in the serviceability limit state, or another solution with a simultaneous reduction in CFRP of 20% and HPC of 25% only lost design factored load and ultimate failure load by 9% and 13%, respectively.

The proposed method has advantages regarding economy and safety due to the ability to optimize the strength of materials and prevent sudden failures for retrofitted slabs. In particular, their carrying capacities are also enhanced considerably.

This study will contribute to simplifying the optimization of strengthened structures using FRP-HPC hybrid retrofit systems and promote the applicability of this technique in practice. Nevertheless, further experimental studies concerning differences in the mechanical properties of retrofit systems, concrete substrates, and environmental conditions are recommended to develop the methodology.

## Figures and Tables

**Table 1:** An overview of the different failure modes of continuous RC slabs.

Location	Failure Modes	First Plastic Hinge	Second Plastic Hinge	Third Plastic Hinge	Shear Failure	Failure Type
End span	D-1e	N2e	N1e	Me	-	Ductile
	D-2e	N2e	Me	N1e	-	Ductile
	D-3e	Me	N2e	N1e	-	Ductile
	DB-1e	N2e	N1e	-	N2e	Brittle
	DB-2e	N2e	Me	-	N2e	Brittle
	DB-3ae	Me	-	-	N2e	Brittle
	DB-3be	Me	N2e	-	N2e	Brittle
	B-1e	N2e	-	-	N2e	Brittle
	B-2e	-	-	-	N2e	Brittle
Interior span	D-1i	N1i, N2i	Mi		-	Ductile
	D-2i	Mi	N1i, N2i		-	Ductile
	DB-1i	N1i, N2i	-		N1i, N2i	Brittle
	DB-2i	Mi	-		N1i, N2i	Brittle
	B-1i	-	-		N1i, N2i	Brittle

**Table 2:** Dimensions and material properties of the existing RC slab.

h (mm)	b (mm)	A_s_ (mm^2^)	d (mm)	f’_c_ (MPa)	γ_c_ (kg/m^3^)	f_y_ (MPa)	E_s_ (GPa)
150	900	426	120	30	2400	400	200

**Table 3:** Mechanical properties of the retrofit system.

HPC		CFRP		
t_H_ (mm)	f′_H_ (mm)	t_F_ (mm)	$f_{\mathrm{fu}}^{*}$	E_F_ (GPa)
30	80	1	600	40

**Table 4:** The preliminary calculation for the control slab and retrofit system.

Analysis	Control Slab
Design section capacity	$\phi_{f}M_{n,N}=$17.83 kNm; $\phi_{f}M_{n,P}=$17.83 kNm; $\phi_{v}V_{n}=$73.9 kN
Design factored load, using Equation (27) $w_{u}=\min\left(w_{\mathrm{uM}},w_{\mathrm{uV}}\right)$$w_{u,M}=\min\left(\frac{\phi_{f}M_{n,P}}{C_{m,\mathrm{Pe}}l_{n}^{2}},\frac{\phi_{f}M_{n,N}}{C_{m,N1}l_{n}^{2}},\frac{\phi_{f}M_{n,N}}{C_{m,N2}l_{n}^{2}}\right)$ $w_{u,V}=\min\left(\frac{\phi_{v}V_{n}}{C_{v1}l_{n}^{}};\frac{\phi_{v}V_{n}}{C_{v2}l_{n}^{}}\right)$	$w_{u}=\min\left(23.58;46.76\right)=23.6\;\mathrm{kN}/m$ $w_{u,M}=\min\left(\frac{17.83}{\left(1/14\right)2.75^{2}};\frac{17.83}{\left(1/16\right)2.75^{2}};\frac{17.83}{\left(1/10\right)2.75^{2}}\right)=23.6\;\mathrm{kN}/m$ $w_{u,V}=\min\left(\frac{2\left(73.9\right)}{1\left(2.75\right)};\frac{2\left(73.9\right)}{1.15\left(2.75\right)}\right)=46.8\;\mathrm{kN}/m$
Failure mode	D-2e, as shown in Figure 8
Ultimate failure load, using Equation (4) for D-2e	$w_{f}=\frac{4}{2.75^{2}}\left(17.83+17.83\frac{\left(1/4+1/10-1/16-1/14\right)}{1/10}\right)=29.8\;\mathrm{kN}/m$
Self-weight $w_{c}=\gamma_{c}\mathrm{bh}$	$w_{c}=\left(2400\times 10^{-2}\right)\left(0.9\right)\left(0.15\right)=3.24\;\mathrm{kN}/m$
The moment at the N2e section due to dead load $M_{D,N2e}=C_{m,N2}w_{c}l_{n}^{2}$	$M_{D,N2e}=\frac{1}{10}\left(3.24\right)\left(2.75^{2}\right)=2.45\times 10^{6}\;\mathrm{Nmm}$
$E_{c}=4700\sqrt{f_{c}^{\prime }}$	$E_{c}=4700\sqrt{30}=25,700\;\mathrm{MPa}$
Crack moment at the N2e section, I_cr,N_	$I_{\mathrm{cr},N}=3.45\times 10^{7}{\;\mathrm{mm}}^{4}$
At the N2e section, kd	kd=26.26 mm
Ultimate strength and strain of CFRP, using Equations (17) and (18)	$f_{\mathrm{fu}}=0.95\left(600\right)=570\;\mathrm{MPa}$; $\varepsilon_{\mathrm{fu}}=\frac{570}{40,000}=0.0143$
The existing state of strain at the N2e section using Equation (19)	$\varepsilon_{\mathrm{bi}}=\frac{\left(2.45\times 10^{6}\right)\left(150-26.26\right)}{\left(3.45\times 10^{7}\right)\left(25,700\right)}=0.00034$

**Table 5:** The initial calculation for the retrofitted slab.

Procedure		Retrofitted Slab
1. Estimate thicknesses of:	-CFRP -HPC	t_F_ = 1 mm t_H_ = 30 mm
2. Check HPC strength using Equation (20)		${\;f\;\prime }_{H}\geq {\;f\;\prime }_{H,\min}=\max\left(8.83;12\right)=12\;\mathrm{MPa}$(OK)
3. Calculate the design strain of CFRP at the N2e section, using Equation (21)		$\varepsilon_{\mathrm{fd}}=0.0112\leq 0.9\varepsilon_{\mathrm{fu}}=0.0128$
4. Estimate the neutral axis depth (Revise of c until equilibrium achieved)		the N2e section: $c_{N}=$28.58 mm mid-span section: $c_{P}=$9.84 mm
5. Calculate the strains of CFRP (ε_fe_), concrete (ε_c_), and tension steel (ε_s_) at the N2e section $\varepsilon_{\mathrm{fe},N}=\varepsilon_{\mathrm{cu}}\left(\frac{h-c_{N}}{c_{N}}\right)-\varepsilon_{\mathrm{bi}}\leq \varepsilon_{\mathrm{fd}}$ $\varepsilon_{c,N}=\left(\varepsilon_{\mathrm{fe},N}+\varepsilon_{\mathrm{bi}}\right)\left(\frac{c_{N}}{h-c_{N}}\right)$ $\varepsilon_{s,N}=\left(\varepsilon_{\mathrm{fe},N}+\varepsilon_{\mathrm{bi}}\right)\left(\frac{d-c_{N}}{h-c_{N}}\right)$ and mid-span section $\varepsilon_{\mathrm{fe},P}=\varepsilon_{\mathrm{cu}}\left(\frac{t_{H}-c_{P}}{c_{P}}\right)\leq \varepsilon_{\mathrm{fd}}$ $\varepsilon_{c,P}=\varepsilon_{\mathrm{fe},P}\left(\frac{c_{P}}{t_{H}-c_{P}}\right)$ $\varepsilon_{s,P}=\varepsilon_{c,P}\left(\frac{{d+t}_{H}+t_{F}-c_{P}}{c_{P}}\right)$		the N2e section $\varepsilon_{\mathrm{fe},N}=0.003\left(\frac{150-28.58}{28.58}\right)-0.00034=0.0124>\varepsilon_{\mathrm{fd}}=0.0112$ $\to \varepsilon_{\mathrm{fe},N}=\varepsilon_{\mathrm{fd}}=0.0112$ $\varepsilon_{c,N}=\left(0.0112+0.00034\right)\left(\frac{28.58}{150-28.58}\right)=0.0027<\varepsilon_{\mathrm{cu}}=0.003$ $\varepsilon_{s,N}=\left(0.0112+0.00034\right)\left(\frac{120-28.58}{150-28.58}\right)=0.0087$ mid-span section $\varepsilon_{\mathrm{fe},P}=0.003\left(\frac{30-9.84}{9.84}\right)=0.0061<\varepsilon_{\mathrm{fd}}=0.0112$ $\varepsilon_{c,P}=0.0061\left(\frac{9.84}{30-9.84}\right)=0.003=\varepsilon_{\mathrm{cu}}$ $\varepsilon_{s,P}=0.003\left(\frac{120+30+1-9.84}{9.84}\right)=0.043$
6. Calculate the stress in tension steel and CFRP at the N2e section using Equations (22) and (23)		the N2e section $f_{s,N}=0.0087\left(200,000\right)=1740\;\mathrm{MPa}>f_{y}=400\;\mathrm{MPa}$$\to f_{s,N}=f_{y}=400\;\mathrm{MPa}$ $f_{\mathrm{fe},N}=0.0112\left(40,000\right)=448\;\mathrm{MPa}$ mid-span section $f_{s,P}=0.043\left(200,000\right)=8607\;\mathrm{MPa}>f_{y}=400\;\mathrm{MPa}$ $\to f_{s,P}=f_{y}=400\;\mathrm{MPa}$ $f_{\mathrm{fe},P}=0.0061\left(40,000\right)=245.85\;\mathrm{MPa}$
7. Check the neutral axis depth for force equilibrium ${\varepsilon^{\prime }}_{c}=\frac{1.7{f^{\prime }}_{c}}{E_{c}}$; $\beta_{1}=\frac{{4\varepsilon^{\prime }}_{c}-\varepsilon_{c}}{{6\varepsilon^{\prime }}_{c}-2\varepsilon_{c}}$ $\alpha_{1}=\frac{{3\varepsilon^{\prime }}_{c}\varepsilon_{c}-\varepsilon_{c}^{2}}{{3\beta }_{1}{\varepsilon^{\prime }}_{c}^{2}}$ $c=\frac{A_{s}f_{s}{+A}_{F}f_{\mathrm{fe}}}{\alpha_{s}f_{c}^{\prime }\beta_{1}b}$		the N2e section, because $\varepsilon_{c,N}<\varepsilon_{\mathrm{cu}}$ ${\varepsilon^{\prime }}_{c,N}=\frac{1.7\left(30\right)}{25,700}=0.002$; $\beta_{1,N}=\frac{4\left(0.002\right)-0.0027}{6\left(0.002\right)-2\left(0.0027\right)}=0.808$ $\alpha_{1,N}=\frac{3\left(0.002\right)\left(0.0027\right)-{\left(0.0027\right)}^{2}}{3\left(0.808\right){\left(0.002\right)}^{2}}=0.922$ $c_{N}=\frac{426\left(400\right)+900\left(448\right)}{0.922\left(30\right)\left(0.808\right)\left(900\right)}=28.58\mathrm{mm}$(OK) mid-span section, because $\varepsilon_{c,P}=\varepsilon_{\mathrm{cu}}$ $\beta_{1,P}=\beta_{1}=0.65;{\;\alpha }_{1,P}=\alpha_{1}=0.85$ $c_{P}=\frac{426\left(400\right)+900\left(245.9\right)}{0.85\left(80\right)\left(0.65\right)\left(900\right)}=9.85\;\mathrm{mm}$(OK)
8. Calculate design flexural and shear strengths		
8.1 Calculate flexural strength at the N2e section contributed by steel $M_{\mathrm{ns},N}=A_{s}f_{s,N}\left(d-\frac{\beta_{1,N}c_{N}}{2}\right)$ and CFRP $M_{\mathrm{nf},N}=A_{F}f_{\mathrm{fe},N}\left(h-\frac{\beta_{1,N}c_{N}}{2}\right)$		$M_{\mathrm{ns},N}=\frac{426\left(400\right)}{10^{6}}\left(120-\frac{0.808\left(28.58\right)}{2}\right)=18.5\;\mathrm{kNm}$ $M_{\mathrm{nf},N}=\frac{\left(900\times 1\right)\left(448\right)}{10^{6}}\left(150-\frac{0.808\left(28.58\right)}{2}\right)=56\;\mathrm{kNm}$
8.2 Calculate flexural strength at the mid-span section contributed by steel $M_{\mathrm{ns},P}=A_{s}f_{s,P}\left({d+t}_{H}+t_{F}-\frac{\beta_{1,P}c_{P}}{2}\right)$ and CFRP $M_{\mathrm{nf},P}=A_{F}f_{\mathrm{fe},P}\left(t_{H}-\frac{\beta_{1,P}c_{P}}{2}\right)$		$M_{\mathrm{ns},P}=\frac{426\left(400\right)}{10^{6}}\left(120+30+1-\frac{0.65\left(9.85\right)}{2}\right)=25.2\;\mathrm{kNm}$ $M_{\mathrm{nf},P}=\frac{\left(900\times 1\right)\left(245.9\right)}{10^{6}}\left(30-\frac{0.65\left(9.85\right)}{2}\right)=5.9\;\mathrm{kNm}$
8.3 Calculate the design flexural strength using Equation (25)		the N2e section $\phi_{f}M_{n,N}=0.9\left[18.5+0.85\left(56\right)\right]=59.5\;\mathrm{kNm}$ mid-span section $\phi_{f}M_{n,P}=0.9\left[25.2+0.85\left(5.9\right)\right]=27.1\;\mathrm{kNm}$
8.4 Calculate the design shear strength using Equation (26)		$\phi_{v}V_{n}=\frac{0.75}{10^{3}}\left(120\sqrt{30}+30\sqrt{80}\right)\frac{900}{6}=104.1\;\mathrm{kN}$
9. Determine design factored load using Equations (27)–(29)		$w_{u}=\min\left(50.1;65.9\right)=50.1\;\mathrm{kN}/m$ $w_{u,M}=\min\left(\frac{27.1}{\left(1/14\right)2.75^{2}};\frac{59.4}{\left(1/16\right)2.75^{2}};\frac{59.4}{\left(1/10\right)2.75^{2}}\right)=50.1\;\mathrm{kN}/m$ $w_{u,V}=\min\left(\frac{2\left(104.1\right)}{1\left(2.75\right)};\frac{2\left(104.1\right)}{1.15\left(2.75\right)}\right)=65.9\;\mathrm{kN}/m$
10. Determine failure mode and failure load		DB-3ae, as shown in Figure 9
		Equation (6), $w_{f}=\frac{2\left(104.1\right)}{1.15\left(2.75\right)}=65.9\;\mathrm{kN}/m$

**Table 6:** Analysis to optimize the retrofit system.

Procedure		R-1 System	R-2 System
1. Estimate thicknesses of:	-CFRP -HPC	$t_{F}=$0.6 mm $t_{H}=$30 mm (keep constant)	$t_{F}=$1 mm $t_{H}=$75 mm
2. Check HPC strength		It is not required to repeat step 2 once t_F_ decreases or t_H_ increases.	
3. Calculate the design strain of CFRP		$\varepsilon_{\mathrm{fd}}=$0.0128	$\varepsilon_{\mathrm{fd}}=$0.0112
4. Estimate the neutral axis depth		$c_{N}=$23 mm $c_{P}=$8.44 mm	$c_{N}=$28.58 mm $c_{P}=$13.8 mm
5. Calculate the strains of CFRP (ε_fe_), concrete (ε_c_), and tension steel (ε_s_)		$\varepsilon_{\mathrm{fe},N}=$0.0128, $\varepsilon_{c,N}=$0.0024, $\varepsilon_{s,N}=$0.0101	$\varepsilon_{\mathrm{fe},N}=$0.0112, $\varepsilon_{c,N}=$0.0027, $\varepsilon_{s,N}=$0.0087
		$\varepsilon_{\mathrm{fe},P}=$0.0077, $\varepsilon_{c,P}=$0.003	$\varepsilon_{\mathrm{fe},P}=$0.0112, $\varepsilon_{c,P}=$0.0025
		$\varepsilon_{s,P}=$0.0505	$\varepsilon_{s,P}=$0.0334
6. Calculate the stress in tension steel and CFRP		$f_{s,N}=$400 MPa, $f_{\mathrm{fe},N}=$513 MPa $f_{s,P}=$400 MPa, $f_{\mathrm{fe},P}=$306.5MPa	$f_{s,N}=$400 MPa, $f_{\mathrm{fe},N}=$449.1 MPa $f_{s,P}=$400 MPa, $f_{\mathrm{fe},P}=$449.1 MPa
7. Check the neutral axis depth for force equilibrium		$c_{N}=$22.99 mm (OK) $c_{P}=$8.44 mm (OK)	$c_{N}=$28.58 mm (OK) $c_{P}=$13.8 mm (OK)
8. Calculate design flexural and shear strengths		$\phi_{f}M_{n,N}=$46.9 kNm $\phi_{f}M_{n,P}=$26.1 kNm	$\phi_{f}M_{n,N}=$59.4 kNm $\phi_{f}M_{n,P}=$50.9 kNm
		$\phi_{v}V_{n}=$104.1 kN	$\phi_{v}V_{n}=$149.4 kN
9. Determine design factored load		$w_{u}=$48.2 kN/m	$w_{u}=$78.6 kN/m
10. Determine failure mode and failure load		D-3e, as shown in Figure 10 Equation (5), $w_{f}=$60.9 kN/m	D-2e, as shown in Figure 11a Equation (4), $w_{f}=$94.9 kN/m
11. Check the moments ratio		$\left(\phi_{f}M_{n,P}/\phi_{f}M_{n,N}\right)$= 0.55 (Not good)	$\left(\phi_{f}M_{n,P}/\phi_{f}M_{n,N}\right)$= 0.86 (Not good)
Adjust iteratively thicknesses of CFRP and HPC to achieve ductile failure mode and desirable moment ratio		It can be achieved with $t_{F}=$0.37 mm and $t_{H}=$30 mm; $w_{u}=$46.7 kN/m; Failure mode D-3e, as shown in Figure 10; $w_{f}=$54 kN/m; $(\phi_{f}M_{n,P}/\phi_{f}M_{n,N})=$25.2/36 = 0.7 (OK)	Not available if t_F_ is kept constant in this case. It can be achieved with $t_{F}=$0.8 mm and $t_{H}=$56 mm; $w_{u}=$71.7 kN/m; Failure mode D-3e, as shown in Figure 11b; $w_{f}=$83 kN/m; $(\phi_{f}M_{n,P}/\phi_{f}M_{n,N})=$38.7/55.3 = 0.7 (OK)

**Table 7:** Summary results of the strengthened slab using optimized versus non-optimized retrofit systems.

Slabs	Failure Mode	$w_{u}$ (kN/m)		$w_{f}$ (kN/m)		$\phi_{f}M_{n,P}$ (kNm)		$\phi_{f}M_{n,N}$ (kNm)		$t_{F}$ (mm)		$t_{H}$ (mm)	
Existing slab	D-2e	23.6		29.8		17.8		17.8					
Retrofit with R-1	D-3e	48.2	[100%]	60.9	[100%]	26.1	[100%]	46.9	[100%]	0.60	[100%]	30	[100%]
Retrofit with optimized R-1	D-3e	46.7	[97%]	54.0	[89%]	25.2	[97%]	36.0	[77%]	0.37	[62%]	30	[100%]
Retrofit with R-2	D-2e	78.6	[100%]	94.9	[100%]	50.9	[100%]	59.4	[100%]	1.00	[100%]	75	[100%]
Retrofit with optimized R-2	D-3e	71.7	[91%]	83.0	[87%]	38.7	[76%]	55.3	[93%]	0.80	[80%]	56	[75%]

## Data Availability

Not applicable.

## Appendix A

Limit equations to divide specific regions corresponding to failure modes for continuous RC slabs were derived according to the recommendations of the ACI committee [7,40]. The basis for establishing these equations is based on appropriate mechanistic analyses detailed in the available literature [30,31] and validated by experimental works [21,28,29]. For the end span, limit equations in each region are determined from the expressions.

(A1) $$M_{n,\mathrm{Ne}}=\frac{{2C}_{m,N2}}{C_{v2}}V_{n}l_{\mathrm{en}}$$

(A2) $$M_{n,\mathrm{Pe}}=\frac{{2C}_{m,\mathrm{Pe}}}{C_{v2}}V_{n}l_{\mathrm{en}}$$

(A3) $$M_{N1e}=\frac{{2C}_{m,N1}}{C_{v2}}V_{n}l_{\mathrm{en}}$$

(A4) $$\frac{M_{n,\mathrm{Ne}}}{M_{n,\mathrm{Pe}}}=\frac{C_{m,N1}}{C_{m,\mathrm{Pe}}}$$

(A5) $$\frac{M_{n,\mathrm{Ne}}}{M_{n,\mathrm{Pe}}}=\frac{C_{m,N2}}{C_{m,\mathrm{Pe}}}$$

(A6) $$M_{n,\mathrm{Pe}}+M_{n,\mathrm{Ne}}\left(\frac{C_{v2}/8+C_{m,N1}-C_{m,\mathrm{Pe}}-C_{v2}C_{m,N1}}{C_{m,N2}}+C_{v2}-1\right)=\frac{1}{4}V_{n}l_{\mathrm{en}}$$

(A7) $$M_{n,\mathrm{Pe}}\left(2C_{v2}-1\right)+M_{n,\mathrm{Ne}}\left(\frac{C_{v2}/4+C_{m,\mathrm{Pe}}-C_{m,N1}-2C_{v2}C_{m,\mathrm{Pe}}}{C_{m,N2}}+1\right)=\frac{1}{2}V_{n}l_{\mathrm{en}}$$

(A8) $$M_{n,\mathrm{Pe}}\left(\frac{C_{v2}/8-C_{m,N2}}{C_{m,\mathrm{Pe}}}\right)+M_{n,\mathrm{Ne}}=\frac{1}{4}V_{n}l_{\mathrm{en}}$$

(A9) $$M_{n,\mathrm{Pe}}\left(\frac{C_{v2}/4+C_{m,N2}-C_{m,N1}-2C_{v2}C_{m,N2}}{C_{m,\mathrm{Pe}}}\right)+2C_{v2}M_{n,\mathrm{Ne}}=\frac{1}{2}V_{n}l_{\mathrm{en}}$$

For interior span, the limit equations for each region are derived from the formulas as follows:

(A10) $$M_{n,\mathrm{Ni}}=\frac{{2C}_{m,N}}{C_{v1}}V_{n}l_{\mathrm{in}}$$

(A11) $$M_{n,\mathrm{Pi}}=\frac{{2C}_{m,\mathrm{Pi}}}{C_{v1}}V_{n}l_{\mathrm{in}}$$

(A12) $$\frac{M_{n,\mathrm{Ni}}}{M_{n,\mathrm{Pi}}}=\frac{C_{m,N}}{C_{m,\mathrm{Pi}}}$$

(A13) $$M_{n,\mathrm{Ni}}\left(\frac{C_{v1}/8-C_{m,\mathrm{Pi}}}{C_{m,\mathrm{Ni}}}\right)+M_{n,\mathrm{Pi}}=\frac{1}{4}V_{n}l_{\mathrm{in}}$$

(A14) $$M_{n,\mathrm{Ni}}+M_{n,\mathrm{Pi}}\left(\frac{C_{v1}/8-C_{m,N}}{C_{m,\mathrm{Pi}}}\right)=\frac{1}{4}V_{n}l_{\mathrm{in}}$$

## Nomenclature

A_s_	Tensile steel area
A_F_	Tensile FRP area
b	Width of RC slab
c	Distance from extreme compression fiber to the neutral axis
C_E_	Environmental reduction factor
C_m_, C_v_	Moment and shear coefficients
d	Distance from the extreme fiber of the compression zone to the center of the steel
d_f_	Distance from the extreme fiber of the compression zone to FRP
E_c_	Elastic modulus of concrete
E_F_	Elastic modulus of CFRP
E_s_	Elastic modulus of steel
f’_c_	Compressive strength of concrete
f’_H_	Compressive strength of the concrete overlay
f_fe_	Effective stress in FRP
f_fu_	Design ultimate strength of FRP
$f_{\mathrm{fu}}^{*}$	Ultimate tensile strength of FRP material, reported by the manufacture
f_s_	Stress in tension steel
f_y_	Yield stress of steel
h	Height of RC slab
I_cr_	Cracked moment of a section
k	The ratio of the depth of the neutral axis to reinforcement depth measured from extreme compression fiber
*l*_en_	Clear span length of the end span
*l*_in_	Clear span length of the interior span
n	Number of CFRP layer
M_n_	Moment carrying capacity
M_n,Ne_, M_n,Pe_	Moment carrying capacities of the support and mid-span sections on the end-span
M_n,Ni_, M_n,Pi_	Moment carrying capacities of the support and mid-span sections on the interior span
M_ns_	Flexural strength contributed by tensile steel
M_nf_	Flexural strength contributed by CFRP	
M_D,N2e_	The moment carrying of the N-2e section
M_u_	The factored moment at a section
t_F_	The thickness of CFRP laminate
t_H_	The thickness of the concrete overlay
V_n_	The shear carrying capacity
V_u_	Factored shear at a section
w_f_	The ultimate failure load
w_u_	The design factored load
w_uM_, w_uV_	Design factored load governed by moment and shear carrying capacities
*ϕ*_f_, *ϕ*_v_	Strength reduction factors for the flexural and shear strength
*ψ*_f_	Strength reduction factors of FRP
α_1_, β_1_	Stress block factors
γ_c_	Unit weight of concrete
ε_bi_	The existing state strain of FRP installation
ε_cu_	The ultimate strain of concrete
ε_fd_	Debonding strain of FRP
ε_fe_	Strain of FRP
ε_fu_	The ultimate strain of FRP
ε_s_	The strain of tensile steel
